# Identification of core genes and outcome in gastric cancer using bioinformatics analysis

**DOI:** 10.18632/oncotarget.20082

**Published:** 2017-08-09

**Authors:** Chenhua Sun, Qi Yuan, Dongdong Wu, Xiaohu Meng, Baolin Wang

**Affiliations:** ^1^ The Second Clinical Medical College of Nanjing Medical University, Nanjing, Jiangsu, China; ^2^ Department of Endocrinology, Huai’an First People’s Hospital, Nanjing Medical University, Huai’an, Jiangsu, China; ^3^ Department of General Surgery, The Second Affiliated Hospital of Nanjing Medical University, Nanjing, Jiangsu, China

**Keywords:** gastric cancer, bioinformatics analysis, protein-protein interaction, diagnosis, combination medicine

## Abstract

Gastric cancer (GC) is a common malignant neoplasm of gastrointestinal tract. We chose gene expression profile of GSE54129 from GEO database aiming to find key genes during the occurrence and development of GC. 132 samples, including 111 cancer and 21 normal gastric mucosa epitheliums, were included in this analysis. Differentially expressed genes (DEGs) between GC patients and health people were picked out using GEO2R tool, then we performed gene ontology (GO) analysis and Kyoto Encyclopedia of Gene and Genome (KEGG) pathway analysis using The Database for Annotation, Visualization and Integrated Discovery (DAVID). Moreover, Cytoscape with Search Tool for the Retrieval of Interacting Genes (STRING) and Molecular Complex Detection (MCODE) plug-in was utilized to visualize protein-protein interaction (PPI) of these DEGs. There were 971 DEGs, including 468 up-regulated genes enriched in focal adhesion, ECM-receptor interaction and PI3K-Akt signaling pathway, while 503 down-regulated genes enriched in metabolism of xenbiotics and drug by cytochrome P450, chemical carcinogenesis, retinol metabolism and gastric acid secretion. Three important modules were detected from PPI network using MCODE software. Besides, Fifteen hub genes with high degree of connectivity were selected, including BGN, MMP2, COL1A1, and FN1. Moreover, the Kaplan–Meier analysis for overall survival and correlation analysis were applied among those genes. In conclusion, this bioinformatics analysis demonstrated that DEGs and hub genes, such as BGN, might promote the development of gastric cancer, especially in tumor metastasis. In addition, it could be used as a new biomarker for diagnosis and to guide the combination medicine of gastric cancer.

## INTRODUCTION

Gastric cancer (GC) is one of the most common tumor of gastrointestinal tract. Lately, the therapy of gastric cancer patients depended largely on the pathologic staging, while pathological biopsy still played an important role during the diagnose of GC. However, this method had no effect on healthy people who were potentially becoming GC patients, even caused the patient unnecessary suffering, and most people with specific discomfort symptoms were advanced patients with gastric cancer [1]. So the global five-year survival rate of gastric cancer was only about 10% [1]. However, in America, this rate was about 30.4% [2], and 5-year survival was over 65% in South Korea because of the extensive screening work for gastric cancer [3].

Recently, several biomarkers were used for the screening and diagnosis of gastric cancer. For example, carbohydrate antigen 72-4 (CA72-4) is one of these tumor markers and it can be used as a screening test for gastric cancer. CA72-4 was an independent significant prognostic factor for relapse free survival and overall survival of gastric cancer [4]. However, this indicator is overly sensitive and it can be elevated in many types of tumors, such as ovarian carcinoma, lung cancer and pancreatic cancer [5–7]. The sensitivity at the time of primary diagnosis of ovarian carcinoma was up to 47% for CA72-4 [5]. The area under the curve of CA72-4 was 0.884 for lung cancer [6] and the sensitivity of CA72-4 in pancreatic cancer was 25.5% [7]. Therefore, it was necessary to investigate the occurrence and development of gastric cancer and detect novel and specific early diagnosis markers.

High throughput sequencing is increasingly being widely used and it has been used as a very significant tool for life sciences, such as cancer early diagnosis, cancer grading and prognosis prediction [8]. In this analysis, we chose GSE54129 from Gene Expression Omnibus (GEO), and used GEO2R online tool to detect the differentially expressed genes (DEGs). Followed by, we established PPI network of the DEGs and picked out hub genes with high degree of connectivity. Moreover, analysis of biological process (BP), molecular function (MF), cellular component (CC) and KEGG pathways of the DEGs and three modules were performed. Besides, overall survival (OS) analysis of these hub genes were made using the Kaplan Meier plotter online database (http://kmplot.com/analysis/) [9]. Then, the correlation analysis based on TCGA database was performed to visualize the potential relationship between genes, and guide the specific medicine treatment for patients.

## RESULTS

### Identification of DEGs and hub genes

There were 111 gastric cancer samples and 21 normal samples in this study. The GEO2R online analysis tool was applied to detect the DEGs, using adjust *P* value < 0.05 and |logFC| ≥ 2 as cut-off criteria. A total of 971 differential expressed genes were detected after the analysis of GSE54129; of which, 468 were up-regulated genes and 503 were down-regulated. Besides, 15 hub genes with high degree of connectivity were selected (Table 1).

**Table 1:** Top 15 hub genes with higher degree of connectivity

Gene	Degree of connectivity	Adjusted *P* value
CTNNB1	24	3.13E-22
FN1	16	5.13E-21
FYN	15	1.98E-34
MMP9	14	2.28E-09
COL1A1	11	1.47E-33
ITCH	11	1.62E-30
TGFB1	10	6.43E-42
THBS1	9	1.03E-22
MMP2	9	3.07E-18
ACTA2	8	1.67E-08
ITGA5	8	5.44E-20
BMP2	8	5.61E-24
BGN	7	1.50E-28
HSPA4	7	7.44E-32
PIK3R1	7	2.29E-37

### GO function and KEGG pathway enrichment analysis

For a more in-depth understanding of the selected DEGs, GO function and KEGG pathway enrichment analysis was applied using DAVID. We imported all DEGs to DAVID software and GO analysis results showed that up-regulated DEGs and down-regulated DEGs were particularly enriched in biological processes (BP), including vasculature development, blood vessel morphogenesis and cardiovascular and circulatory system development for up-regulated DEGs, and for down-regulated including epithelial cell differentiation, digestion and epithelium development (Table 2). For molecular function (MF), the upregulated DEGs were enriched in glycosaminoglycan, heparin, sulfur compound, receptor and protein complex binding, and the down-regulated DEGs were enriched in oxidoreductase activity, acting on CH-OH group of donors, NAD or NADP as acceptor and retinol dehydrogenase and aldo-keto reductase (NADP) activity (Table 2). In addition, GO cell component (CC) analysis also displayed that the up-regulated DEGs were significantly enriched in the extracellular matrix, proteinaceous extracellular matrix and extracellular region part, and down-regulated DEGs enriched in extracellular region part and apical part of cell (Table 2).

**Table 2:** Gene ontology analysis of differentially expressed genes associated with gastric cancer

Expression	Category	Term	Count	%	*P-*Value	FDR
Up-regulated	GOTERM_BP_FAT	GO:0001944∼vasculature development	67	19.65	7.50E-31	1.43E-27
	GOTERM_BP_FAT	GO:0001568∼blood vessel development	62	18.18	5.72E-28	1.09E-24
	GOTERM_BP_FAT	GO:0048514∼blood vessel morphogenesis	56	16.42	2.00E-26	3.82E-23
	GOTERM_BP_FAT	GO:0072358∼cardiovascular system development	75	21.99	1.28E-25	2.43E-22
	GOTERM_BP_FAT	GO:0072359∼circulatory system development	75	21.99	1.28E-25	2.43E-22
	GOTERM_MF_FAT	GO:0005539∼glycosaminoglycan binding	28	8.21	3.99E-15	6.14E-12
	GOTERM_MF_FAT	GO:0008201∼heparin binding	23	6.74	4.66E-13	7.16E-10
	GOTERM_MF_FAT	GO:1901681∼sulfur compound binding	26	7.62	3.41E-12	5.23E-09
	GOTERM_MF_FAT	GO:0005102∼receptor binding	64	18.77	6.71E-10	1.03E-06
	GOTERM_MF_FAT	GO:0032403∼protein complex binding	42	12.32	2.71E-09	4.17E-06
	GOTERM_CC_FAT	GO:0031012∼extracellular matrix	66	19.35	8.53E-32	1.23E-28
	GOTERM_CC_FAT	GO:0005578∼proteinaceous extracellular matrix	52	15.25	7.99E-28	1.16E-24
	GOTERM_CC_FAT	GO:0044421∼extracellular region part	159	46.63	7.12E-21	1.03E-17
	GOTERM_CC_FAT	GO:0005615∼extracellular space	87	25.51	9.70E-20	1.40E-16
	GOTERM_CC_FAT	GO:0005576∼extracellular region	171	50.15	4.15E-18	6.00E-15
Down-regulated	GOTERM_BP_FAT	GO:0030855∼epithelial cell differentiation	42	10.85	2.99E-13	5.61E-10
	GOTERM_BP_FAT	GO:0007586∼digestion	21	5.43	5.83E-11	1.10E-07
	GOTERM_BP_FAT	GO:0060429∼epithelium development	49	12.66	3.66E-08	6.88E-05
	GOTERM_BP_FAT	GO:0006805∼xenobiotic metabolic process	14	3.62	5.49E-08	1.03E-04
	GOTERM_BP_FAT	GO:0006629∼lipid metabolic process	56	14.47	7.06E-08	1.33E-04
	GOTERM_MF_FAT	GO:0016614∼oxidoreductase activity, acting on CH-OH group of donors	22	5.68	2.20E-13	3.43E-10
	GOTERM_MF_FAT	GO:0016616∼oxidoreductase activity, acting on the CH-OH group of donors, NAD or NADP as acceptor	17	4.39	9.66E-10	1.51E-06
	GOTERM_MF_FAT	GO:0004745∼retinol dehydrogenase activity	8	2.07	2.60E-08	4.05E-05
	GOTERM_MF_FAT	GO:0004033∼aldo-keto reductase (NADP) activity	7	1.81	1.08E-05	0.016818
	GOTERM_MF_FAT	GO:0016709∼oxidoreductase activity, acting on paired donors, with incorporation or reduction of molecular oxygen, NAD(P)H as one donor, and incorporation of one atom of oxygen	7	1.81	1.71E-04	0.266413
	GOTERM_CC_FAT	GO:0044421∼extracellular region part	124	32.04	5.03E-08	7.04E-05
	GOTERM_CC_FAT	GO:0005576∼extracellular region	140	36.18	1.05E-07	1.47E-04
	GOTERM_CC_FAT	GO:0045177∼apical part of cell	26	6.72	1.38E-07	1.93E-04
	GOTERM_CC_FAT	GO:0070062∼extracellular exosome	92	23.77	3.26E-06	0.004567
	GOTERM_CC_FAT	GO:1903561∼extracellular vesicle	92	23.77	4.04E-06	0.005651

GO: Gene Ontology; FDR: False Discovery Rate.

Table 3 showed the most significantly enriched KEGG pathway of the up-regulated and down-regulated DEGs. The up-regulated DEGs were enriched in focal adhesion, ECM-receptor interaction, PI3K-Akt signaling pathway, protein digestion and absorption and vascular smooth muscle contraction, while the down-regulated DEGs were enriched in metabolism of xenbiotics by cytochrome P450, chemical carcinogenesis, retinol metabolism, drug metabolism by cytochrome P450 and gastric acid secretion.

**Table 3:** KEGG pathway analysis of differentially expressed genes associated with gastric cancer

Category	Term	Count	%	*P*-Value	Genes	FDR
Up-regulated DEGs	hsa04510:Focal adhesion	25	7.3	5.06E-11	TLN1, TNC, MYL9, COL6A3, COL6A2, COL6A1, ZYX, THBS1, COL11A1, THBS2, PIK3R1, SPP1, THBS4, FN1, COL4A2, COL4A1, IGF1, FLNA, VEGFC, FYN, ITGA5, ITGA7, COL1A2, COL1A1, MYLK	6.32E-08
	hsa04512:ECM-receptor interaction	16	4.7	1.13E-09	COL4A2, COL4A1, TNC, ITGA5, ITGA7, COL6A3, COL6A2, COL1A2, COL6A1, COL1A1, THBS1, THBS2, COL11A1, SPP1, THBS4, FN1	1.42E-06
	hsa04151:PI3K-Akt signaling pathway	25	7.3	1.34E-06	MCL1, OSMR, TNC, BCL2L1, IL7R, COL6A3, COL6A2, COL6A1, IL2RG, THBS1, COL11A1, THBS2, PIK3R1, SPP1, THBS4, FN1, COL4A2, COL4A1, IGF1, YWHAE, VEGFC, ITGA5, ITGA7, COL1A2, COL1A1	0.001667
	hsa04974:Protein digestion and absorption	12	3.5	5.64E-06	COL4A2, COL14A1, COL4A1, COL6A3, ELN, COL1A2, COL6A2, COL12A1, COL6A1, COL1A1, COL11A1, COL10A1	0.007038
	hsa04270:Vascular smooth muscle contraction	12	3.5	9.93E-05	EDNRA, GNA13, ACTG2, ACTA2, CALD1, PLA2G2A, GUCY1A3, GUCY1B3, CALCRL, MYLK, KCNMB1, MYL9	0.123856
Down-regulated DEGs	hsa00980:Metabolism of xenobiotics by cytochrome P450	12	3.1	2.08E-07	GSTA1, CYP3A4, CYP3A5, CBR1, CYP2C9, ADH1C, AKR7A3, ADH1A, ADH7, UGT2B15, AKR1C1, ALDH3A1	2.56E-04
	hsa05204:Chemical carcinogenesis	12	3.1	4.70E-07	GSTA1, CYP3A4, CYP3A5, CBR1, CYP2C19, CYP2C9, CYP2C18, ADH1C, ADH1A, ADH7, UGT2B15, ALDH3A1	5.78E-04
	hsa00830:Retinol metabolism	11	2.8	5.47E-07	CYP3A4, RDH12, CYP3A5, CYP2C9, CYP2C18, ADH1C, DHRS9, SDR16C5, ADH1A, ADH7, UGT2B15	6.72E-04
	hsa00982:Drug metabolism - cytochrome P450	11	2.8	8.44E-07	GSTA1, CYP3A4, FMO5, CYP3A5, CYP2C19, CYP2C9, ADH1C, ADH1A, ADH7, UGT2B15, ALDH3A1	0.001037
	hsa04971:Gastric acid secretion	11	2.8	1.66E-06	KCNJ16, KCNJ15, ATP4A, GNAQ, ATP4B, SLC26A7, KCNE2, GAST, CA2, SST, KCNK10	0.002035

KEGG: Kyoto Encyclopedia of Genes and Genomes; FDR: False Discovery Rate.

### The kaplan meier plotter and expression level of hub genes

The prognostic information of the 15 hub genes was freely available in http://kmplot.com/analysis/. It was found that expression of BGN (HR 1.9 [1.56–2.32], *P* = 1.3 × 10^–10^) was associated with worse overall survival (OS) for gastric cancer patients, as well as MMP2 (HR 1.78 [1.47–2.16], *P* = 2.6 × 10^–9^), COL1A1 (HR 1.49 [1.22–1.81], *P* = 8.2 × 10^–5^) and FN1 (HR 1.46 1.23–1.74], *P* = 1.3 × 10^–5^) (Figure 3). Then, we used GEPIA to detect the hub genes expression level between cancer and healthy people, and Figure 4A reflected that compared to normal, BGN significantly increased expression levels in cancer patients.

### Hub genes and module screening from PPI network

**Figure 1:**
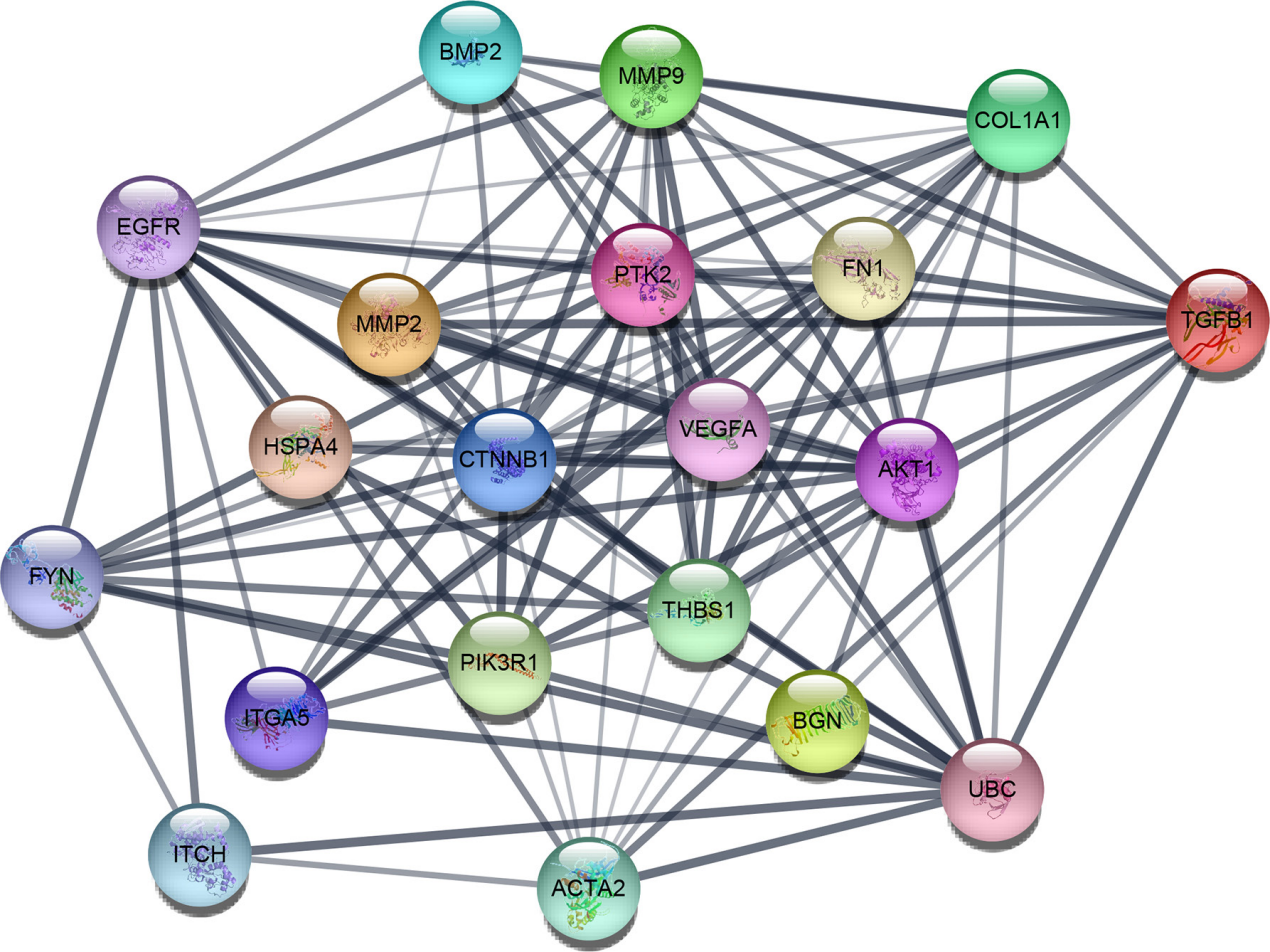
The protein-protein interaction network of top 15 hub genes

Based on the information in the STRING protein query from public databases, we made the PPI network of the top 15 hub genes with higher degree of connectivity (Figure 1). We selected ACTA2, BGN, MMP2, THBS1, TGFB1, COL1A1, FN1 and ITCH, which with worse overall survival situation according to Kaplan Meier plotter. Based on the GO function, KEGG pathway analysis and the survival analysis, we found that BGN, MMP2, COL1A1 and FN1 were enriched in extracellular matrix, especially in proteinaceous extracellular matrix.

**Figure 2:**
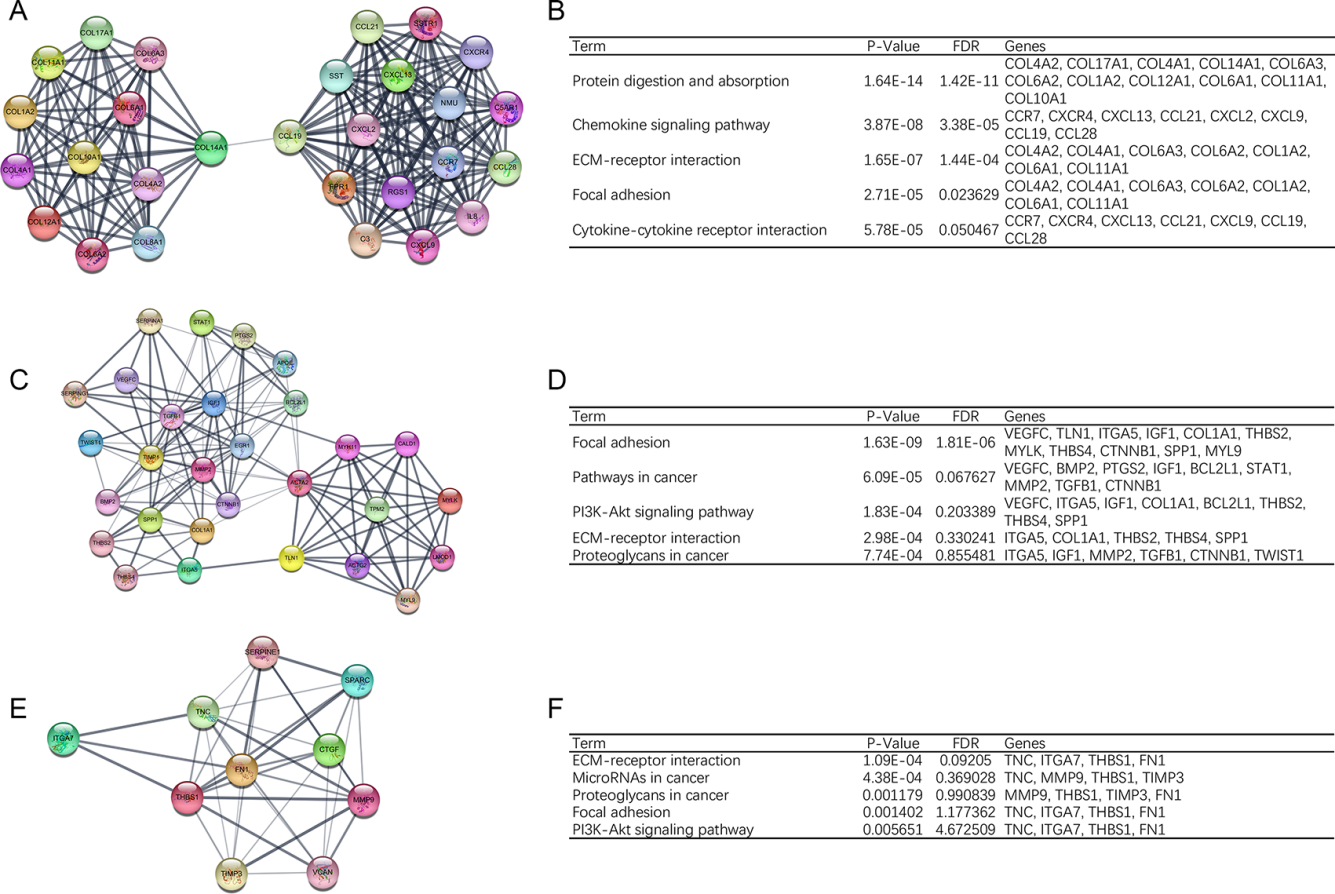
Top 3 modules from the protein-protein interaction network (**A**) module 1, (**B**) the enriched pathways of module 1, (**C**) module 2, (**D**) the enriched pathways of module 2, (**E**) module 3, (**F**) the enriched pathways of module 3.

In order to detect significant modules in this PPI network, we used MCODE plug-in. The top 3 modules were selected (Figure 2). KEGG pathway enrichment analysis showed that these three modules were mainly associated with focal adhesion, PI3K-Akt signaling pathway and ECM-receptor interaction (Figure 2).

## DISCUSSION

Although the decline trends of the GC morbidity have been noted in recent years, it is still the second leading cause of cancer deaths in China [10]. It is mainly because of failure to early screening and diagnosis. Therefore, sensitive and specific biomarkers of gastric cancer are urgently needed to be detected.

In this analysis, 111 gastric cancer samples and 21 normal samples were included from the GEO database of GSE54129. A total of 971 DEGs were screened, including 468 up-regulated genes and 503 down-regulated genes. For a more in-depth understanding of these DEGs, we performed GO function and KEGG pathway analysis of these DEGs.

The GO analysis showed that up-regulated DEGs were mainly involved in vessel morphogenesis, glycosaminoglycan binding and extracellular matrix, and down-regulated DEGs were involved in epithelial cell differentiation, oxidoreductase activity, acting on CH-OH group of donors, extracellular region part and digestion. Furthermore, the KEGG pathways of up-regulated DEGs included focal adhesion, ECM-receptor interaction, PI3K-Akt signaling pathway, protein digestion and absorption and vascular smooth muscle contraction, while the down-regulated DEGs were enriched in metabolism of xenbiotics by cytochrome P450, chemical carcinogenesis, retinol metabolism, drug metabolism by cytochrome P450 and gastric acid secretion. Among these DEGs, 15 hub genes with high degree of connectivity were selected in the PPI network. Four hub genes with worse overall survival (OS) of gastric cancer patients were detected and the Kaplan Meier-plotter was applied to visualize them, including BGN, MMP2, COL1A1 and FN1. Those four hub genes were enriched in extracellular matrix, especially in proteinaceous extracellular matrix. A recent study reported that extracellular matrix could play a vital role in breast cancer metastasis [11]. We could hypothesize that those four genes might contribute to the metastasis of gastric cancer,

BGN, encoded by this gene was a small cell or cell surrounding proteoglycans, which were structurally closely related to two other small proteoglycans, core proteoglycans and fibrin regulatory proteins. BGN took part in blood vessel remodeling, extracellular matrix organization and carbohydrate metabolic process. It exists in the extracellular exosome and this cell component had an inseparable relationship with ACAT1 [12], which inhibited cholesterol esterification in T cells, leading to potentiated effector function and enhanced proliferation of CD8^+^T cells [13]. The Figure 4B showed the results of the correlation analysis between BGN and ACAT1. BGN and ACAT1 are obviously positively correlated. A combined therapy of the ACAT inhibitor avasimibe plus an anti-PD-1 antibody showed a delightful effect of suppressing the tumor development and progression [13], which provided a whole new perspective for us to perform medical work, using specific tumor inhibitor of BGN and an anti-PD-1 antibody to fight with carcinoma.

**Figure 3:**
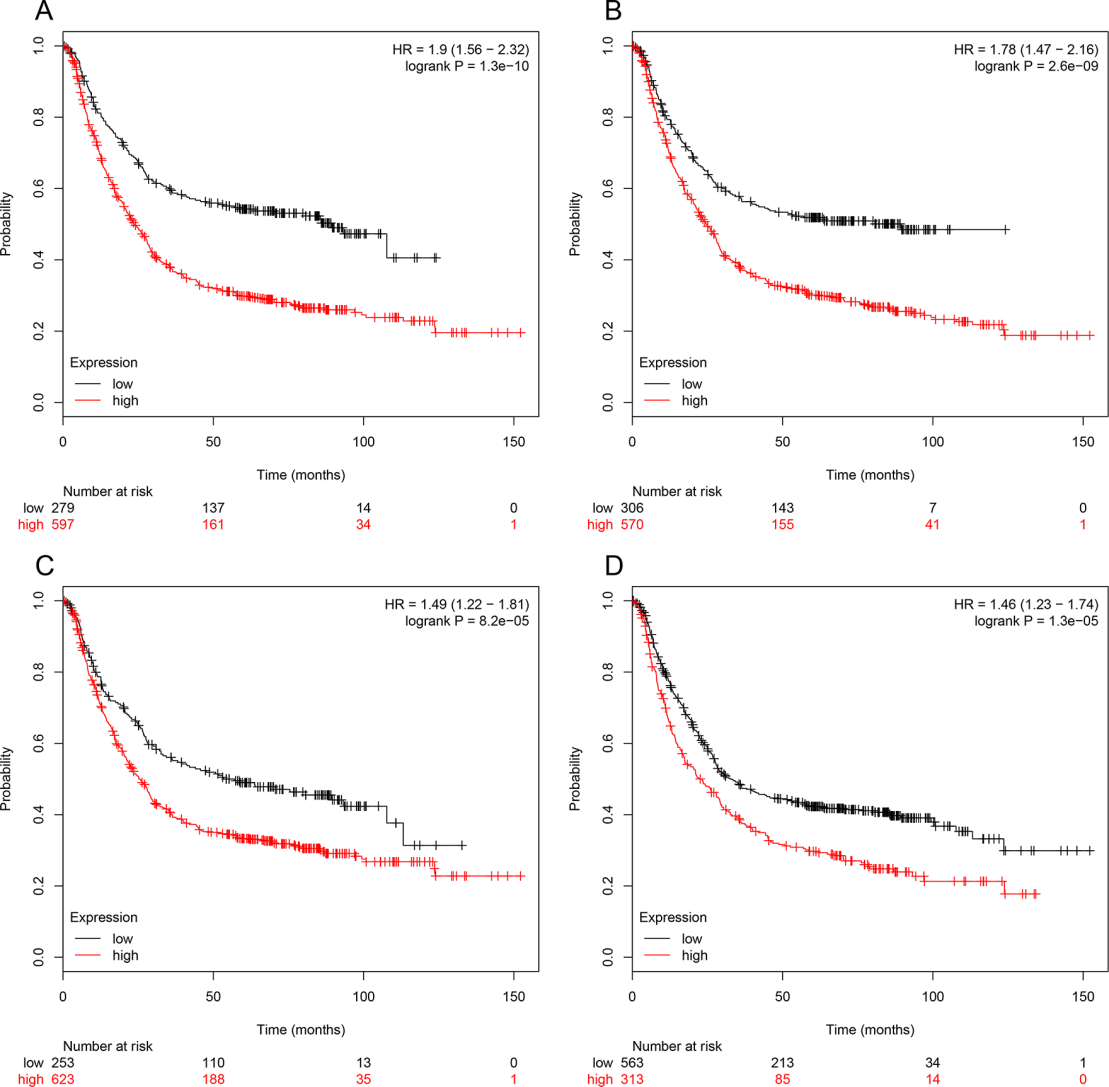
Prognostic value of four genes (BGN (**A**), MMP2 (**B**), COL1A1 (**C**), FN1 (**D**)) in gastric cancer patients. The desired Affymetrix IDs are valid: 201261_x_at(BGN), 201069_at (MMP2), 202311_s_at (COL1A1), 212464_s_at (FN1). HR: hazard ratio, CI: confidence interval.

**Figure 4:**
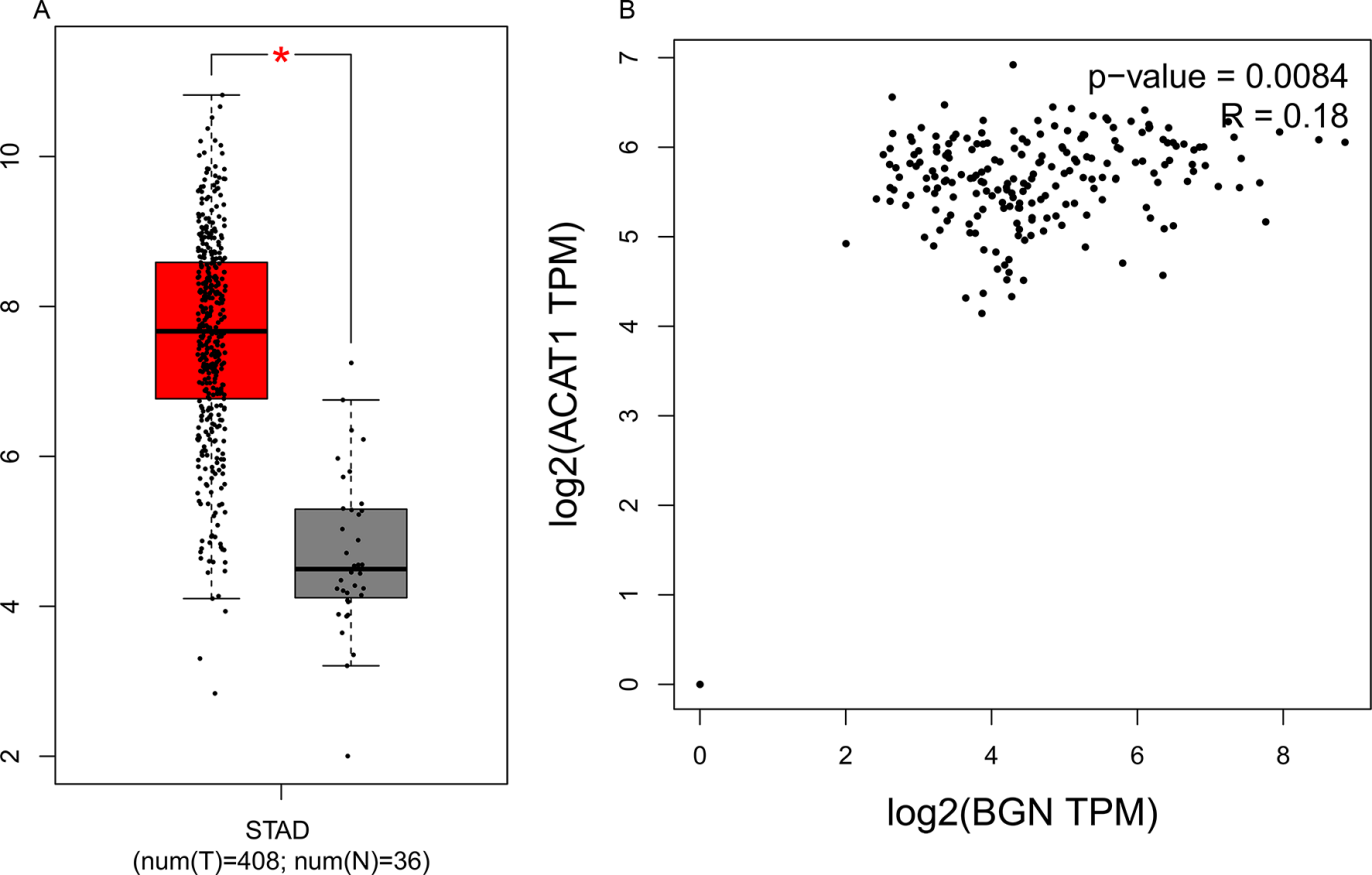
(**A**) Expression level of BGN in cancer and normal tissues. STAD: Stomach Adenocarcinoma; *P < 0.05. (**B**) The correlation analysis between BGN and ACAT1. BGN and ACAT1 are obviously positively correlated.

Matrix metallopeptidase 2 (MMP2), a member of MMP family which were zinc-dependent enzymes capable of cleaving components of molecules and the extracellular matrix involved in signal transduction. Unlike most MMP family members, activation of MMP2 could happen on the cell membrane, and it could be activated by proteases, or with no requirement for proteolytical removal of the pro-domain by its S-glutathiolation. MMP2 was involved in many pathways in cancer, and it existed in many proteoglycans in cancer. Moreover, the expression level of MMP2 in sarcoma was much high than that in normal tissue based on TCGA database.

Analysis of the three selected modules from the PPI network showed that gastric cancer was associated with focal adhesion, PI3K-Akt signaling pathway and ECM-receptor interaction. It was reported that high expression of focal adhesion kinase (FAK) activity was associated with elevated level of fibrosis and poor CD8^+^ T cell infiltration. Focal adhesion kinase inhibition could substantially limit tumor progression and extend the survival time of cancer patients [14]. The top perturbed pathways in gastric cancer included focal adhesion and adherens junction, in which RHOA gene and any other differentially expressed genes we detected participated in these biological processes [15]. Collagen, type I, alpha 1 (COL1A1) participated in the process of focal adhesion, and it could provide a new way for us to perform genome personalized treatment. Several studies indicated that numerous components of the phosphatidylinositol-3-kinase (PI3K)/AKT pathway were targeted by amplification, mutation and translocation more frequently than any other pathway in cancer patients, leading to pathway activation. Fibronectin 1(FN1), predominantly expressed in various malignancies but not in normal tissues, was mainly involved in this pathway [16] and a specific tumor regimen for the FN1 gene could be implemented. The control of cell growth is increased by a coordinated response to growth factor and cell-extracellular matrix (ECM) interactions. To be one of the family of ECM receptor, integrin was critical to the coordinated responses [17]. FN1 was also involved in this pathway and the expression level of FN1 was closely associated with tumor growth and metastasis [18].

All in all, our bioinformatics analysis identified DEGs and they might play a central role in the occurrence, development and prognosis of gastric cancer. In this study, a total of 971 DEGs and 15 hub genes were selected, and BGN, MMP2, COL1A1 and FN1 might be the core genes of gastric cancer. In order to get more accurate correlation results, we need to start a series of verification experiments later to confirm the results of this prediction. Anyway, this study could provide some powerful evidence for the future genomic individualized treatment of gastric cancer.

## MATERIALS AND METHODS

### Microarray data

We chose gene expression profile of GSE54129 from GEO database, which was a public and freely available database. GSE54129, which was based on agilent GPL570 platform ([HG-U133_Plus_2] Affymetrix Human Genome U133 Plus 2.0 Array). The GSE54129 dataset included 132 samples, containing 111 gastric cancer samples and 21 normal gastric mucosa epithelium. We also downloaded the Series Matrix File of GSE54129 from GEO database.

### Data processing of DEGs

GEO2R (https://www.ncbi.nlm.nih.gov/geo/geo2r/) was applied to detect differentially expressed genes between gastric cancer samples and normal samples [19]. GEO2R is an interactive online tool that allows users to compare two or more groups of samples in a GEO Series and it can analyze most GEO series with gene symbol. The adjust *P* values were utilized to reduce the false positive rate using Benjamini and Hochberg false discovery rate method by default. The adjust *P* value < 0.05 and |logFC| ≥ 2 were set as the cut off criterion. Then, 971 DEGs were found, including 468 up-regulated genes and 503 down-regulated genes, and we selected the top 15 genes with high degree of connectivity as hub genes.

### Gene ontology and KEGG pathway analysis of DEGs

Gene ontology analysis (GO) is a common useful method for annotating genes and gene products and for identifying characteristic biological attributes for high-throughput genome or transcriptome data [20]. Kyoto Encyclopedia of Genes and Genomes (KEGG) is a collection of databases dealing with genomes, biological pathways, diseases, drugs, and chemical substances [21]. The Database for Annotation, Visualization and Integrated Discovery (DAVID, https://david.ncifcrf.gov/) is a web-based online bioinformatics resource that aims to provide tools for the functional interpretation of large lists of genes or proteins [22]. *P* < 0.05 was set as the cut-off criterion. We could visualize the core biological processes, molecular functions, cellular components and pathways among those DEGs using DAVID.

### PPI network and module analysis

Search Tool for the Retrieval of Interacting Genes (STRING) is online tool designed to evaluate the protein–protein interaction (PPI) information [23]. To detect the potential relationship among those DEGs, we used STRING app in Cytoscape and mapped the DEGs into STRING. And confidence score ≥ 0.4, maximum number of interactors = 0 were set as the cut off criterion. Moreover, the Molecular Complex Detection (MCODE) app was utilized to screen modules of PPI network in Cytoscape with degree cutoff = 2, node score cutoff = 0.2, k-core = 2, and max. depth = 100. The pathway analysis of genes in each module was performed by DAVID. Also, 15 hub genes were also mapped into STRING with confidence score ≥ 0.4, maximum number of interactors ≤ 5. GO and KEGG pathway analysis were also made to explore the potential information.

### Comparison of the hub genes expression level

The GEPIA (http://gepia.cancer-pku.cn/index.html) a newly developed interactive web server for analyzing the RNA sequencing expression data of 9,736 tumors and 8,587 normal samples from the TCGA and the GTEx projects, using a standard processing pipeline [24]. It provides customizable functions such as tumor and normal differential expression analysis, and we could demonstrate the expression of hub genes in gastric cancer tissues and normal tissues. Then the boxplot was performed to visualize the relationship.

### Survival analysis of hub genes

Kaplan Meier-plotter (KM plotter, http://kmplot.com/analysis/) could assess the effect of 54675 genes on survival using 10,461 cancer samples, including 5143 breast, 1816 ovarian, 2437 lung and 1,065 gastric cancer patients with a mean follow-up of 69, 40, 49 and 33 months [9]. The relapse free and overall survival information were based on GEO (Affymetrix microarrays only), EGA and TCGA database. The hazard ratio (HR) with 95% confidence intervals and log rank *P* value were calculated and displayed on the plot.
